# Supplementary material for: Comprehensive Evidence of Carrier-Mediated Distribution of Amantadine to the Retina across the Blood–Retinal Barrier in Rats
Source: Pharmaceutics. 2021 Aug 26;13(9):1339. doi: 10.3390/pharmaceutics13091339 (PMC8469395; doi:10.3390/pharmaceutics13091339)
Supplement: Supplementary file 1 [file pharmaceutics-13-01339-s001.zip › pharmaceutics-1316225-supplementary.pdf]

pharmaceutics

MDPI

Supplementary Materials: Comprehensive Evidence of Carrier-Mediated Distribution of Amantadine to the Retina across the Blood–Retinal Barrier in Rats

Yusuke Shinozaki, Shin-ichi Akanuma, Yuika Mori, Yoshiyuki Kubo and Ken-ichi Hosoya

Iintracarotid Artery Injection

Following the previous reports of in vivo retinal transport study [1–3], [^{3}H]amantadine (5 µCi/rat) and [^{14}C]*n*-butanol (0.5 µCi/rat), a diffusible BRB-permeable marker, in 200 µL injection buffer (pH 7.4; composed of 144 mM NaCl, 4 mM KCl, 2.8 mM $CaCl_2$, and 10 mM 2-[4-(hydroxyethyl)-1-piperazinyl]ethanesulfonic acid [HEPES]-NaOH) were injected into the common carotid artery. Immediately after the injection (15 sec), retinas were collected and lysed with 2 N NaOH. After adding 2 N HCl for neutralization, the radioactivities of [^{3}H]amantadine or [^{14}C]*n*-butanol were measured using a liquid scintillation counter (LSC 6100, Aloka, Tokyo, Japan).

In Vitro Uptake Reaction

In accordance with established protocols for the uptake study [1–4], the cells were rinsed with extracellular fluid (ECF) buffer (122 mM NaCl, 25 mM $NaHCO_3$, 3 mM KCl, 1.4 mM $CaCl_2$, 1.2 mM $MgSO_4$, 0.4 mM K_2HPO_4, 10 mM D-glucose and 10 mM HEPES, pH 7.4) three times at 37°C. The uptake reaction was started by adding 200 µL ECF buffer containing 0.1 µCi radiolabeled compounds with or without inhibitor at 37°C. This reaction was then terminated by washing the wells three times with ice-cold ECF buffer. After washing, the cells were lysed and then neutralized with 1N NaOH and 1N HCl.

To evaluate extracellular Na^+ or resting membrane potential dependence, the buffer was replaced with equimolar amounts of choline chloride and choline bicarbonate or KCl and $KHCO_3$, respectively. Cl^--free ECF buffer was prepared by replacing equimolar amounts of sodium gluconate, potassium gluconate, and calcium gluconate (gluconate replacement). ECF buffer at pH 6.4 and pH 8.4 was prepared by replacing HEPES with equimolar 2-morpholinoethanesulfonic acid and 2-amino-2-hydroxymethyl-1,3-propanediol, respectively. The intracellular pH was increased and decreased by acute treatment and pretreatment (20 min) of cells with 30 mM NH_4Cl [4]. Measurement of [^{3}H]amantadine- and [^{3}H]verapamil-derived radioactivities was performed using a liquid scintillation counter (SLC-6100, Aloka), and the cellular protein content was measured using a DC protein assay kit (Bio-Rad, Hercules, CA, USA).

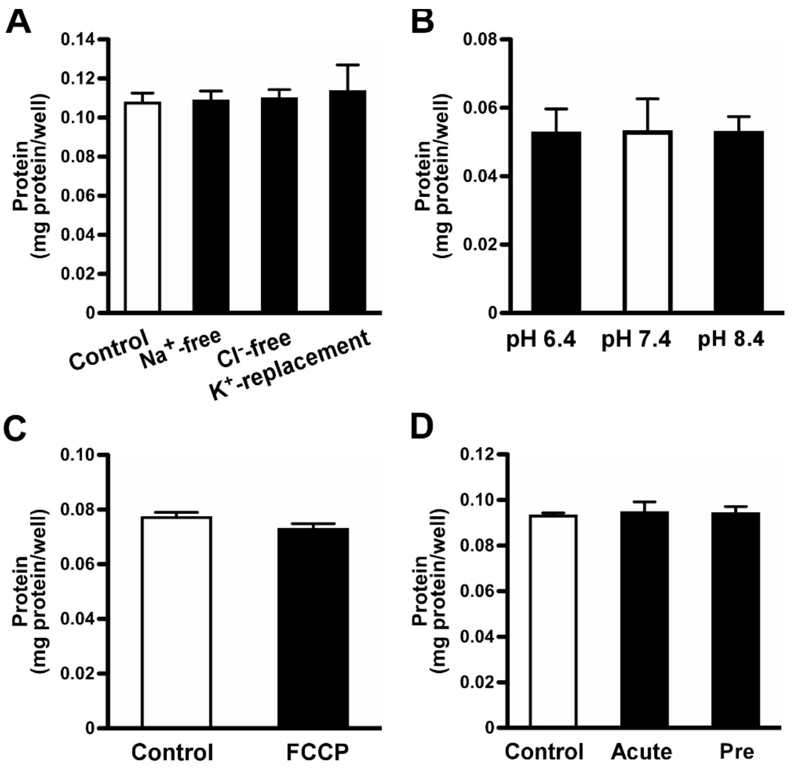

Figure S1. The cellular protein amount in the group of Figure 3. Each column represents the mean ± S.D. (n = 3). In the tested group, the significant difference from the control was not observed (p > 0.05).

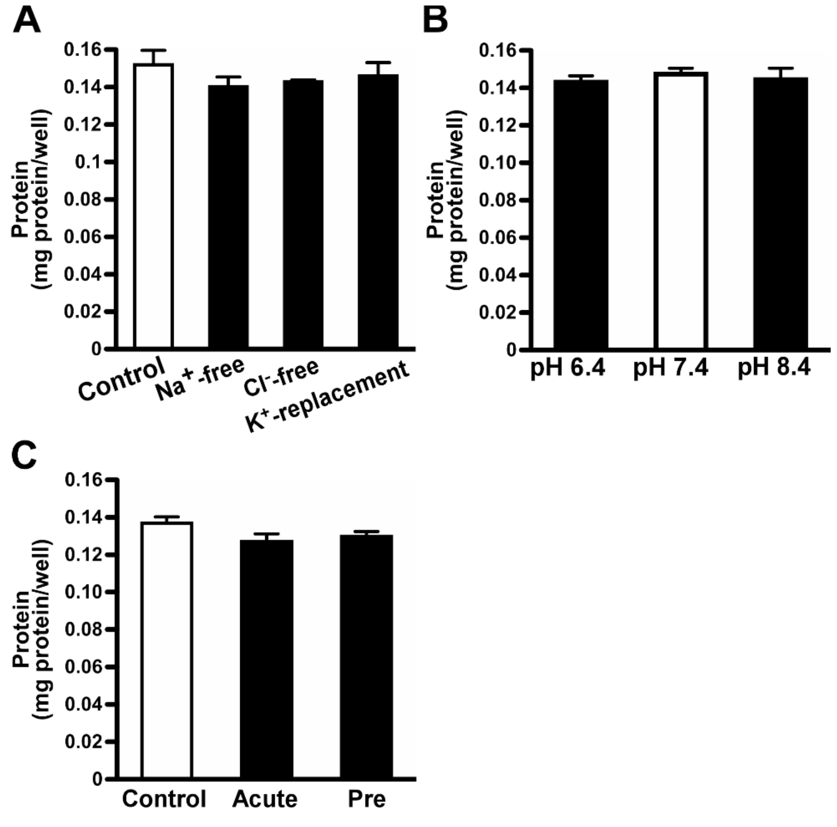

Figure S2. The cellular protein amount in the group of Figure 5. Each column represents the mean ± S.D. (n = 3). In the tested group, the significant difference from the control was not observed (p > 0.05).

References

1. Kubo, Y.; Kusagawa, Y.; Tachikawa, M.; Akanuma, S.; Hosoya, K. Involvement of a novel organic cation transporter in verapamil transport across the inner blood-retinal barrier. *Pharm. Res.* **2013**, *30*, 847-856.
2. Kubo, Y.; Shimizu, Y.; Kusagawa, Y.; Akanuma, S.; Hosoya, K. Propranolol transport across the inner blood-retinal barrier: potential involvement of a novel organic cation transporter. *J. Pharm. Sci.* **2013**, *102*, 3332-3342.
3. Kubo, Y.; Tsuchiyama, A.; Shimizu, Y.; Akanuma, S.; Hosoya, K. Involvement of carrier-mediated transport in the retinal uptake of clonidine at the inner blood-retinal barrier. *Mol. Pharm.* **2014**, *11*, 3747-3753.
4. Tega, Y.; Tabata, H.; Kurosawa, T.; Kitamura, A.; Itagaki, F.; Oshitari, T.; Deguchi, Y. Structural requirements for uptake of diphenhydramine analogs into hCMEC/D3 Cells via the proton-coupled organic cation antiporter. *J. Pharm. Sci.* **2021**, *110*, 397-40.
